# Supplementary material for: Disrupting USP39 deubiquitinase function impairs the survival and migration of multiple myeloma cells through ZEB1 degradation
Source: J Exp Clin Cancer Res. 2024 Dec 30;43:335. doi: 10.1186/s13046-024-03241-2 (PMC11686864; doi:10.1186/s13046-024-03241-2)
Supplement: Supplementary file 1 — Supplementary Material 1. [file 13046_2024_3241_MOESM1_ESM.pdf]

Figure S1

CD138 staining

A

Bone marrow of healthy individuals

B

Bone marrow of MM patients

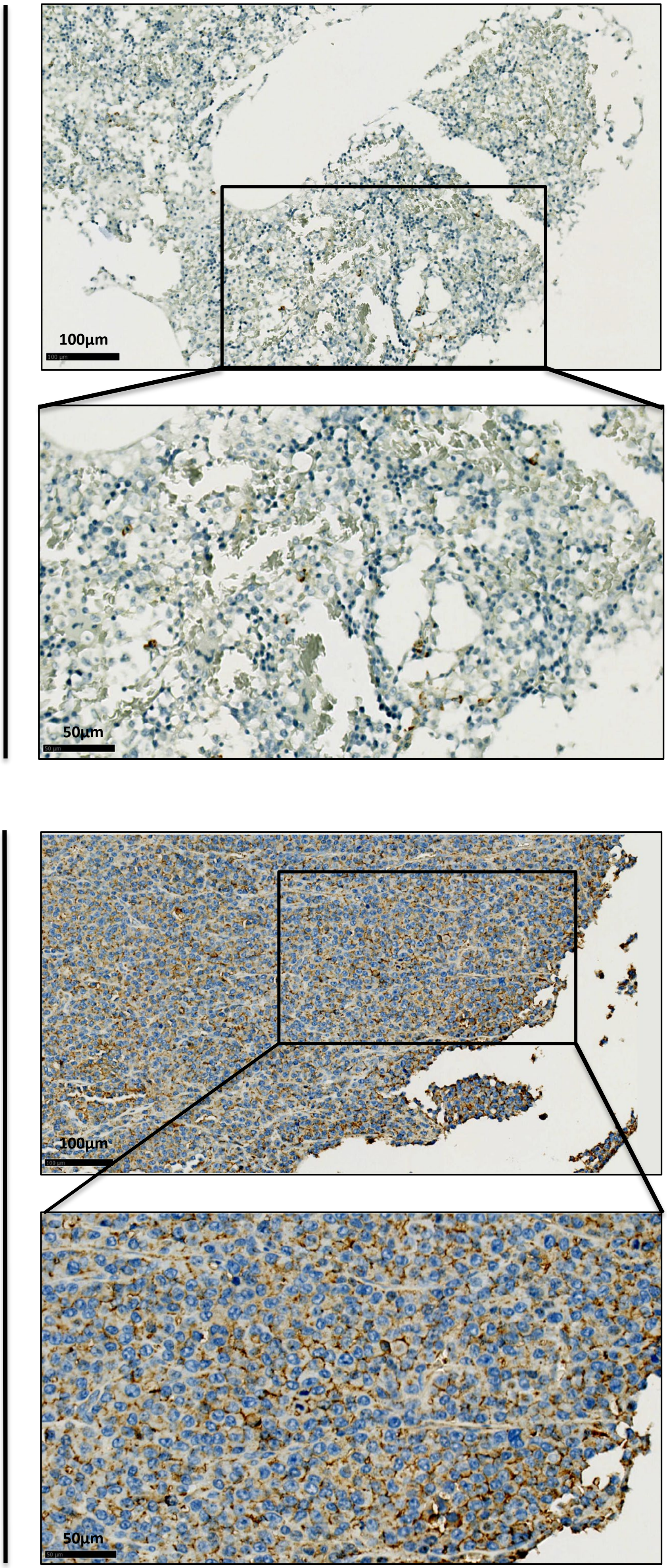

Figure S2

A

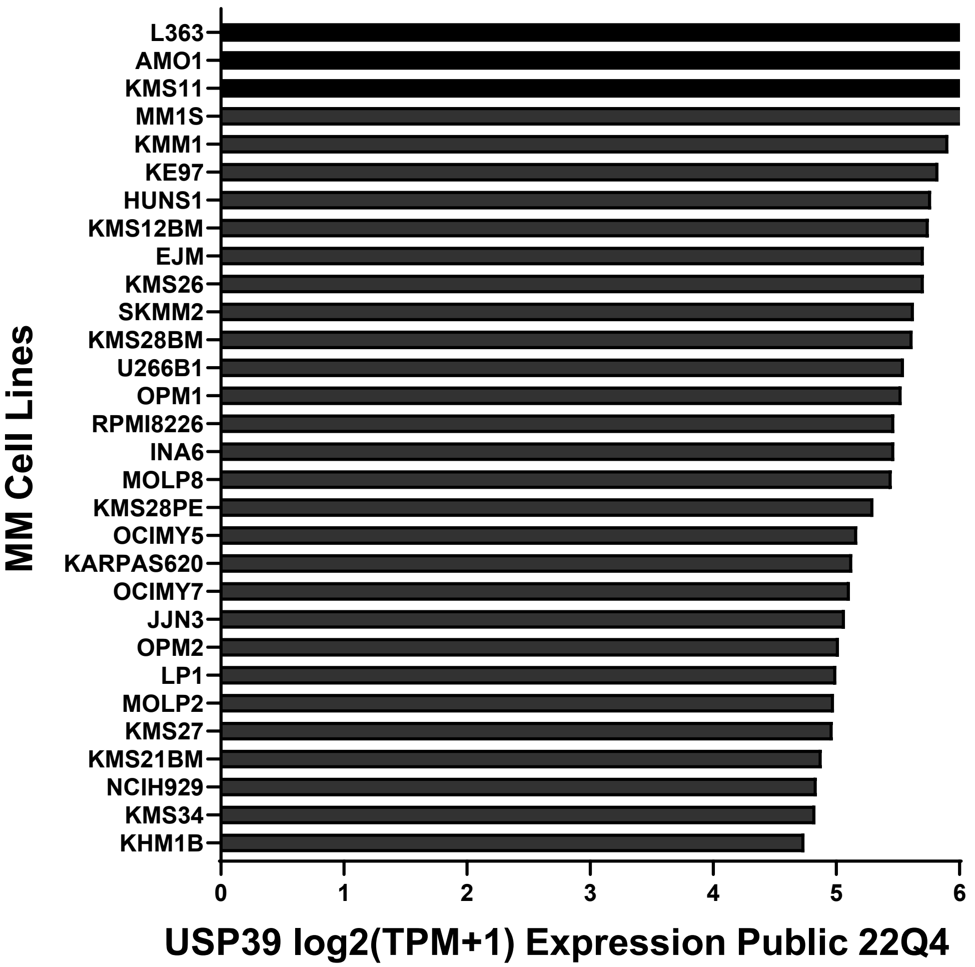
L363
AMO1
KMS11
MM1S
KMM1
KE97
HUNS1
KMS12BM
EJM
KMS26
SKMM2
KMS28BM
U266B1
OPM1
RPMI8226
INA6
MOLP8
KMS28PE
OCIMY5
KARPAS620
OCIMY7
JJN3
OPM2
LP1
MOLP2
KMS27
KMS21BM
NCIH929
KMS34
KHM1B
MM Cell Lines
0
1
2
3
4
5
6
USP39 log2(TPM+1) Expression Public 22Q4

B

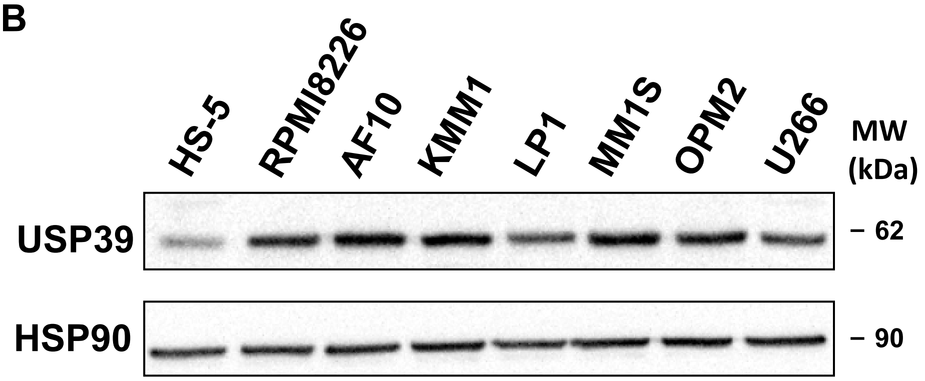
HS-5
RPMI8226
AF10
KMM1
LP1
MM1S
OPM2
U266
MW (kDa)
USP39
– 62
HSP90
– 90

Figure S3

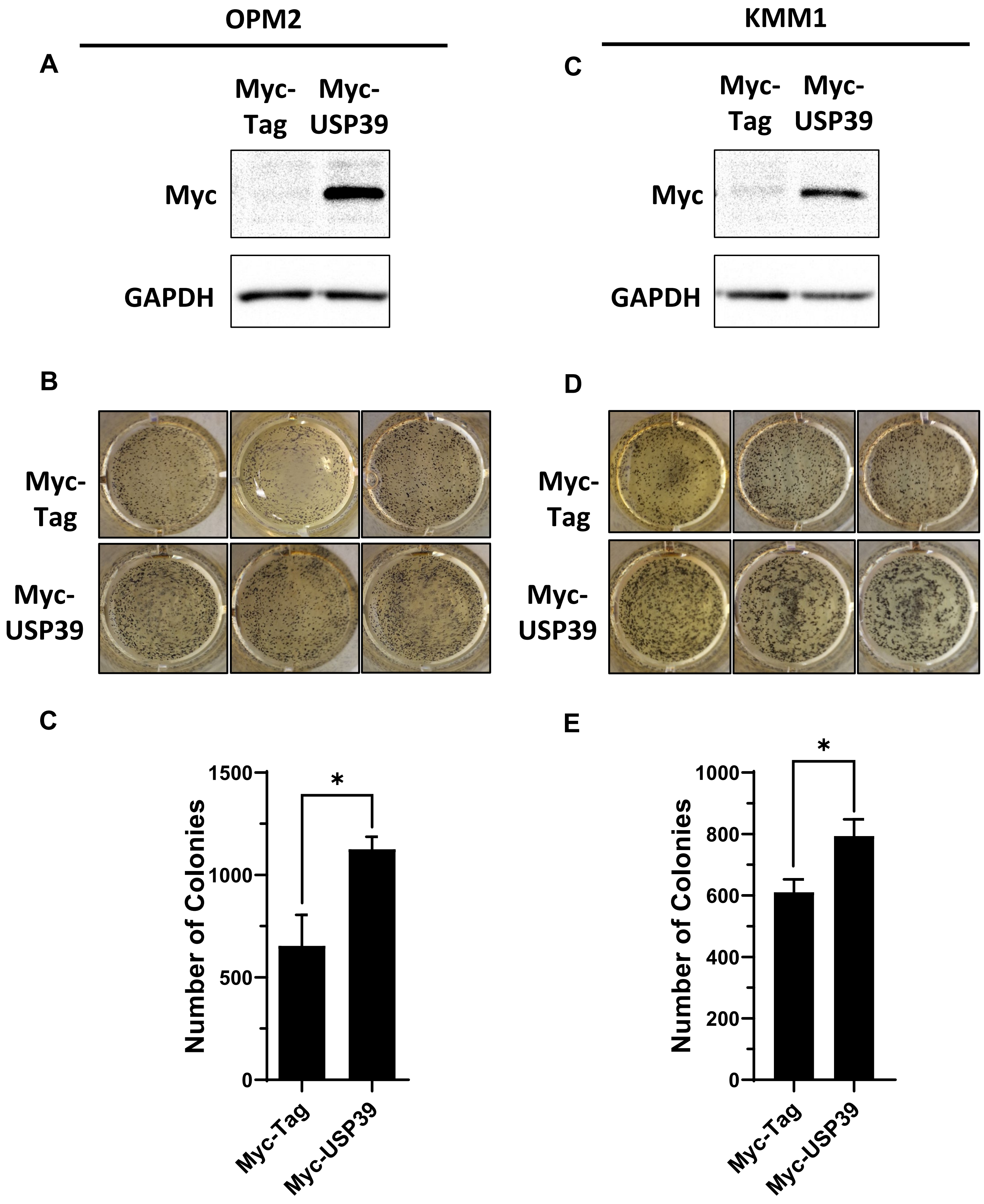

OPM2
KMM1
A
C
Myc-
Tag
Myc-
USP39
Myc
GAPDH
B
D
Myc-
Tag
Myc-
USP39
C
E
Number of Colonies
1500
1000
500
0
*
Myc-Tag
Myc-USP39
1000
800
600
400
200
0

Figure S4

A

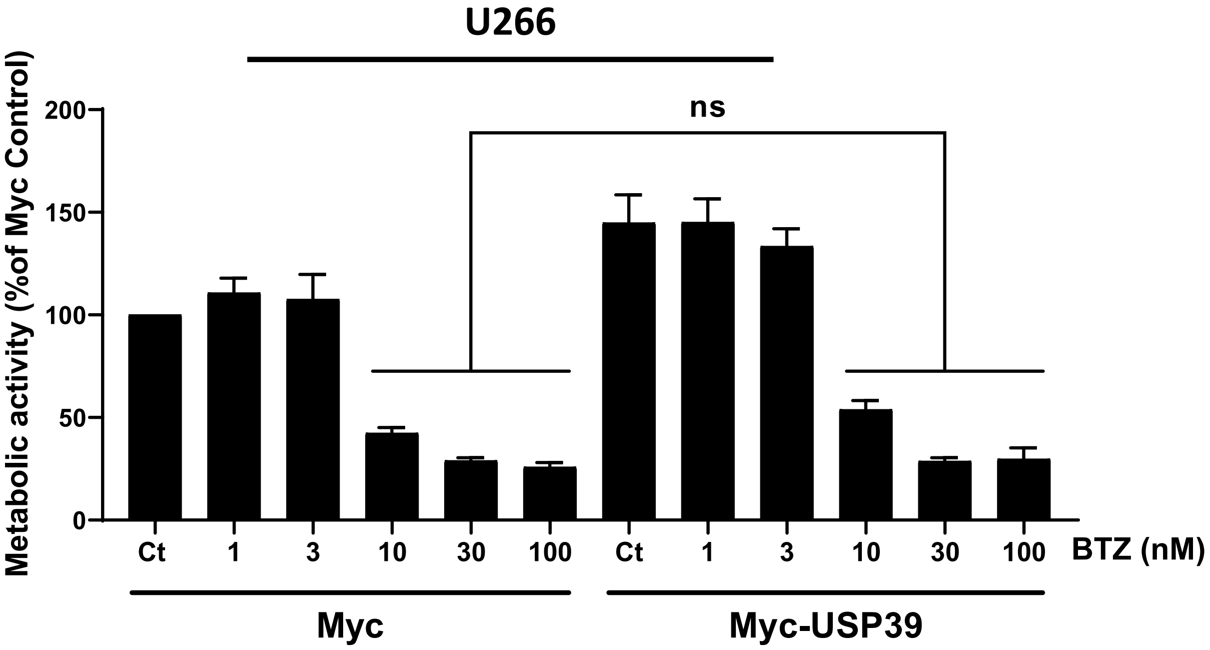
U266
Metabolic activity (%of Myc Control)
200
150
100
50
0
ns
Ct
1
3
10
30
100
Ct
1
3
10
30
100
BTZ (nM)
Myc
Myc-USP39

B

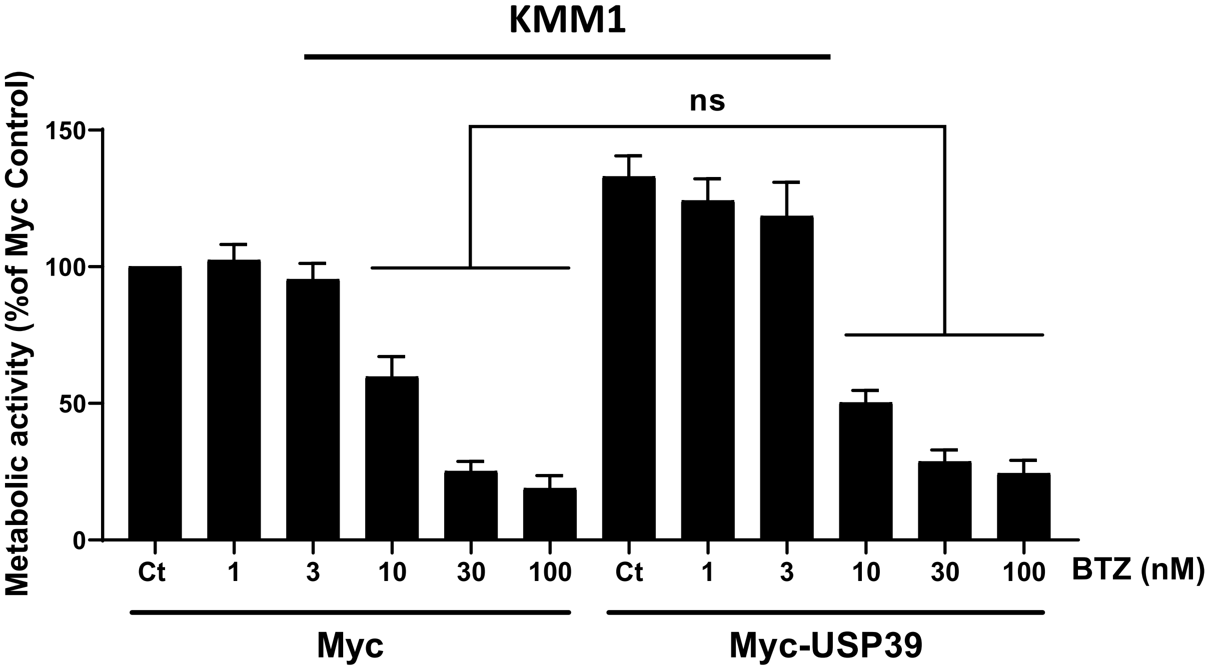
KMM1
Metabolic activity (%of Myc Control)
150
100
50
0
ns
Ct
1
3
10
30
100
Ct
1
3
10
30
100
BTZ (nM)
Myc
Myc-USP39

Figure S5

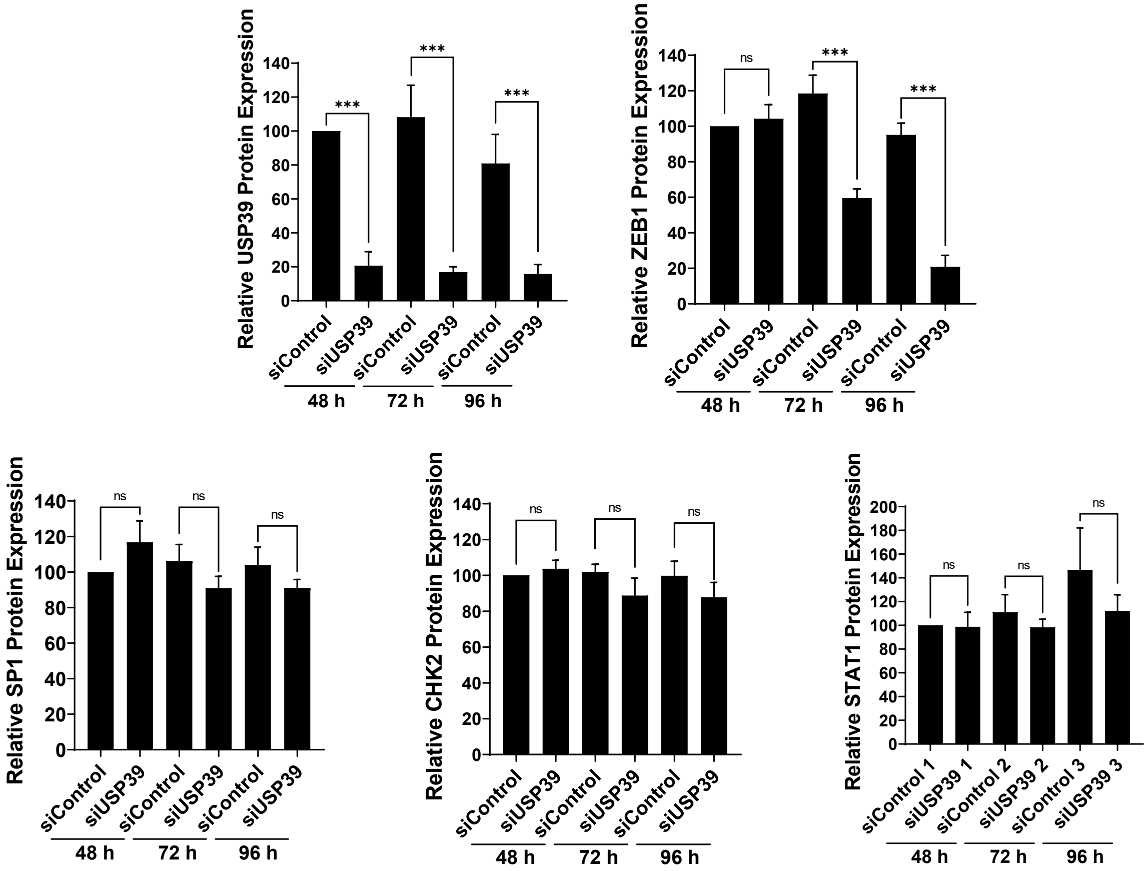

Relative USP39 Protein Expression
140
120
100
80
60
40
20
0
***
siControl
siUSP39
48 h
72 h
96 h
Relative ZEB1 Protein Expression
ns
Relative SP1 Protein Expression
Relative CHK2 Protein Expression
Relative STAT1 Protein Expression
200
180
160
siControl 1
siUSP39 1
siControl 2
siUSP39 2
siControl 3
siUSP39 3

Figure S6

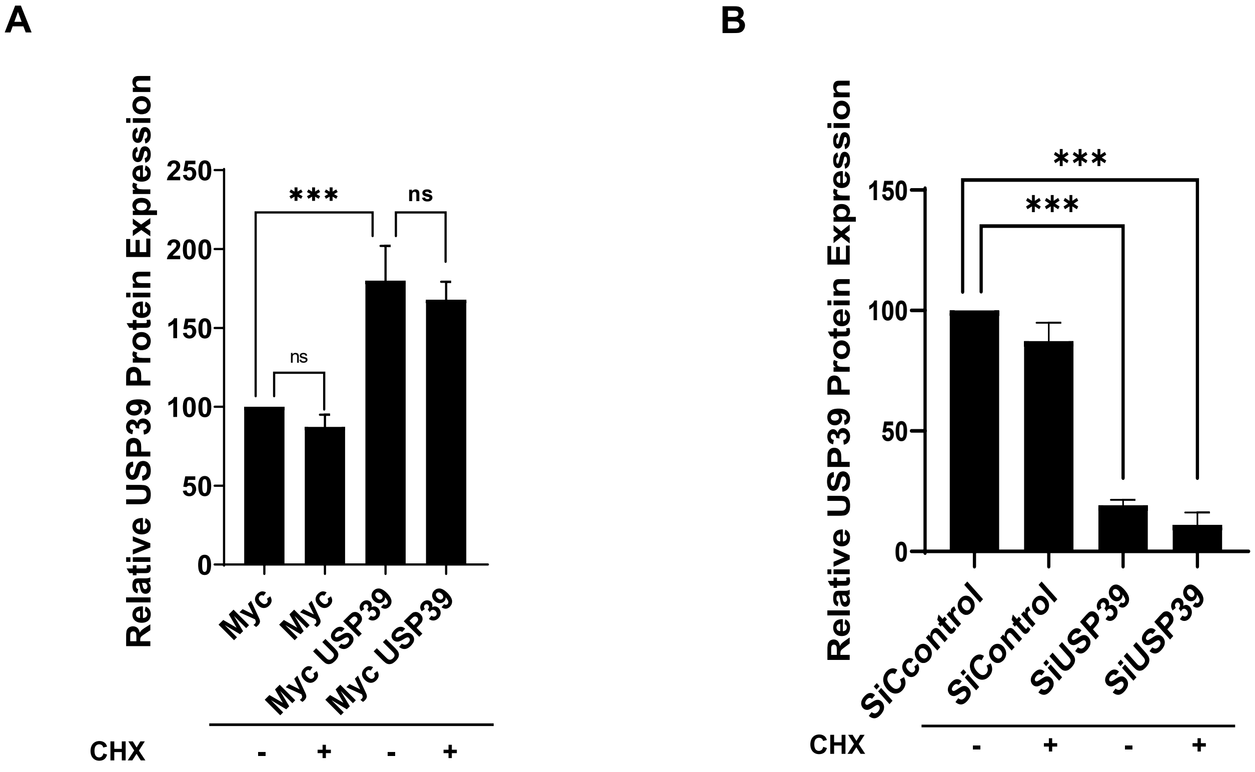
A
Relative USP39 Protein Expression
250
200
150
100
50
0
***
ns
ns
Myc
Myc
Myc USP39
Myc USP39
CHX
-
+
-
+
B
Relative USP39 Protein Expression
150
100
50
0
***
***
SiCcontrol
SiControl
SiUSP39
SiUSP39
CHX
-
+
-
+

Figure S7

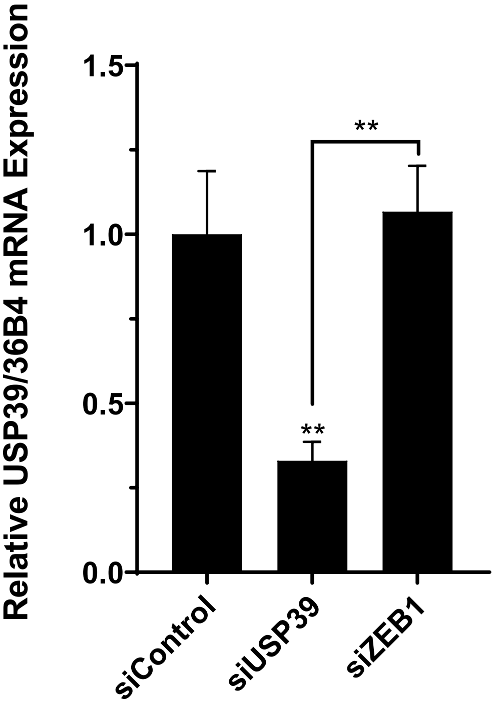

Relative USP39/36B4 mRNA Expression
1.5
1.0
0.5
0.0
**
**
siControl
siUSP39
siZEB1

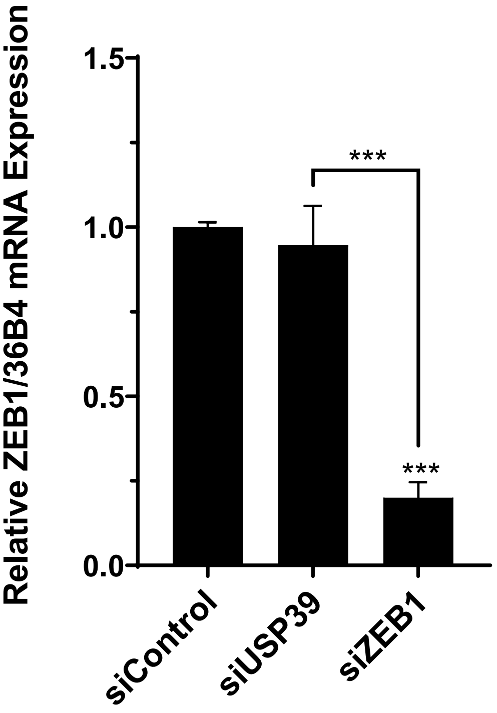

Relative ZEB1/36B4 mRNA Expression
1.5
1.0
0.5
0.0
***
***
siControl
siUSP39
siZEB1

Figure S8

A

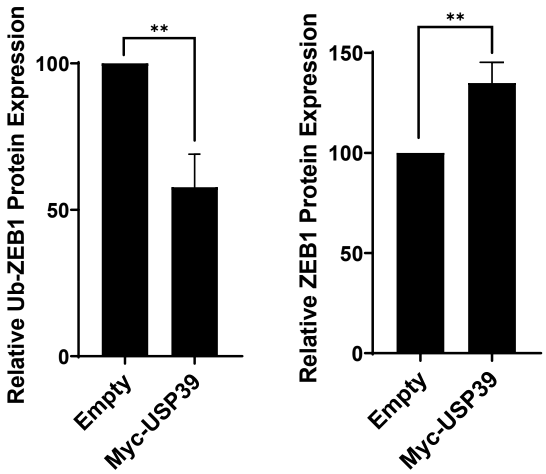
Relative Ub-ZEB1 Protein Expression
**
100
50
0
Empty
Myc-USP39
Relative ZEB1 Protein Expression
**
150
100
50
0
Empty
Myc-USP39

B

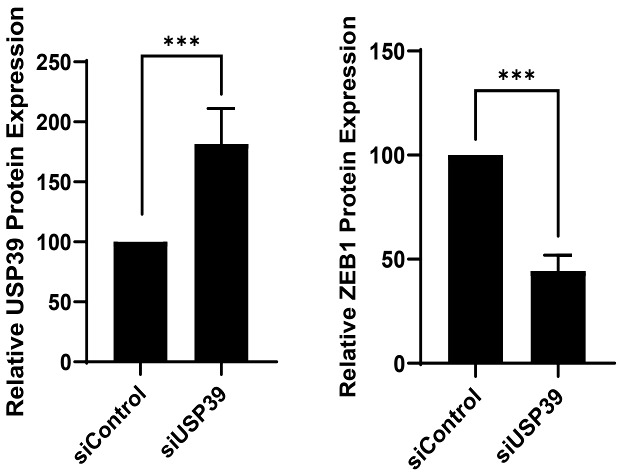
Relative USP39 Protein Expression
***
250
200
150
100
50
0
siControl
siUSP39
Relative ZEB1 Protein Expression
***
150
100
50
0
siControl
siUSP39

Figure S9

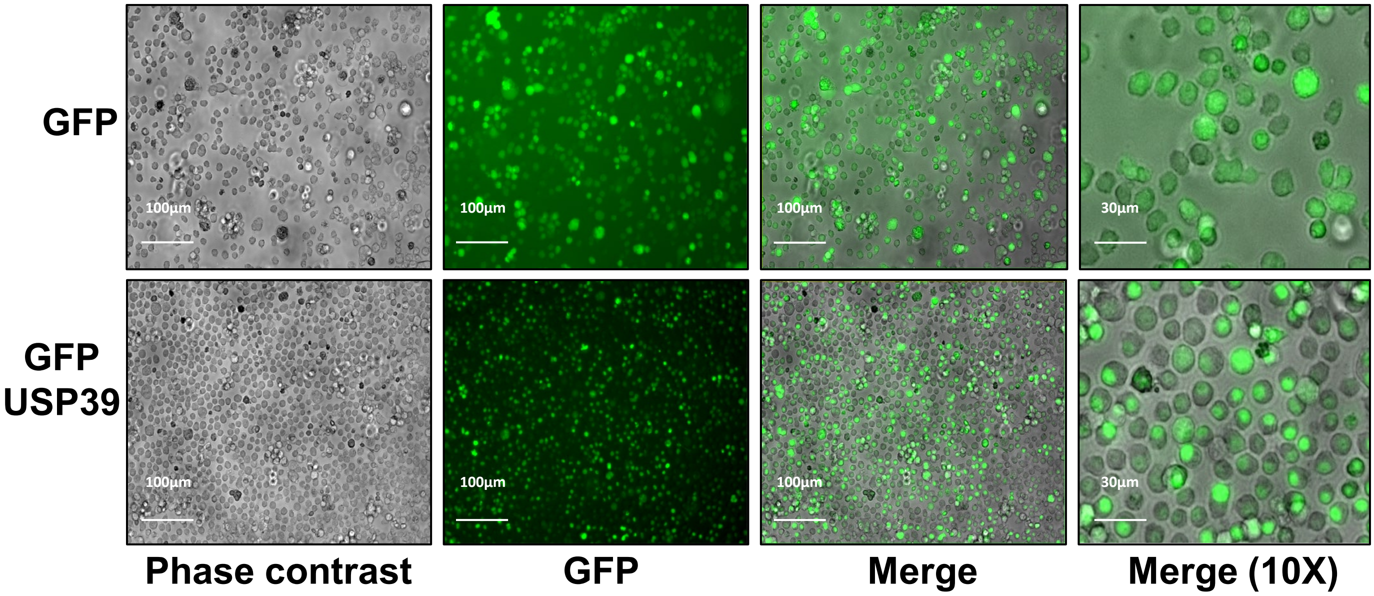

Figure S10

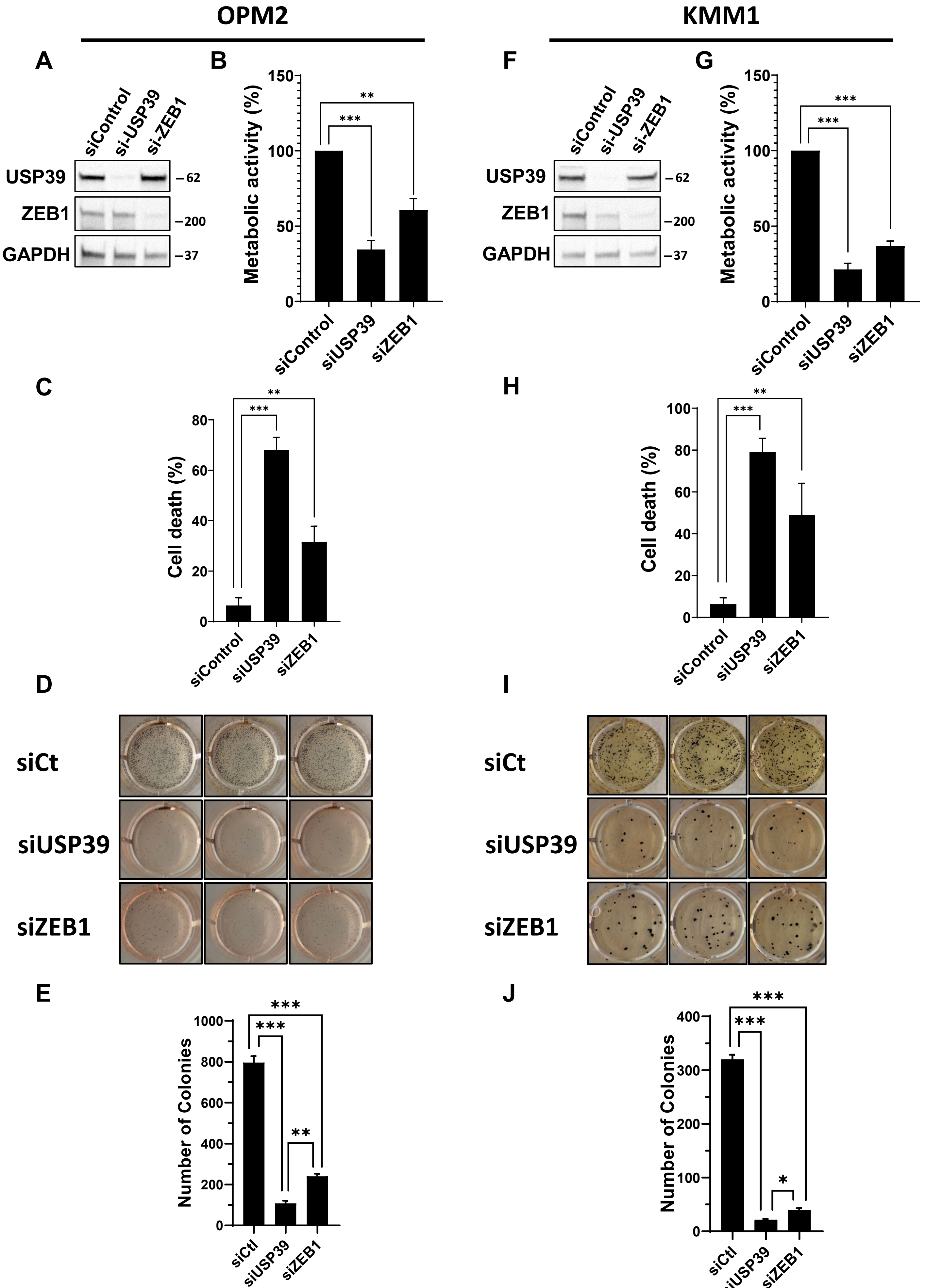

OPM2
KMM1
A
B
C
D
E
F
G
H
I
J
siControl
si-USP39
si-ZEB1
USP39
ZEB1
GAPDH
–62
–200
–37
Metabolic activity (%)
Cell death (%)
Number of Colonies
siControl
siUSP39
siZEB1
siCt
siUSP39
siZEB1
siCtl
**
***
*
0
50
100
150
20
40
60
80
200
400
600
800
1000
300

Figure S11

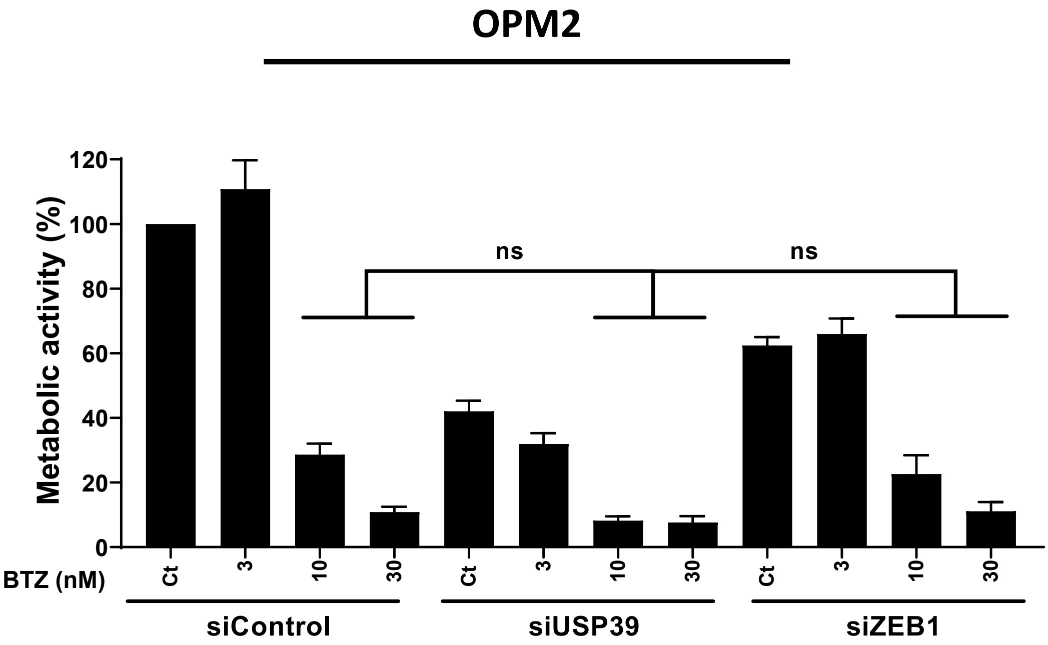
OPM2
Metabolic activity (%)
120
100
80
60
40
20
0
ns
ns
BTZ (nM)
Ct
3
10
30
Ct
3
10
30
Ct
3
10
30
siControl
siUSP39
siZEB1

Figure S12

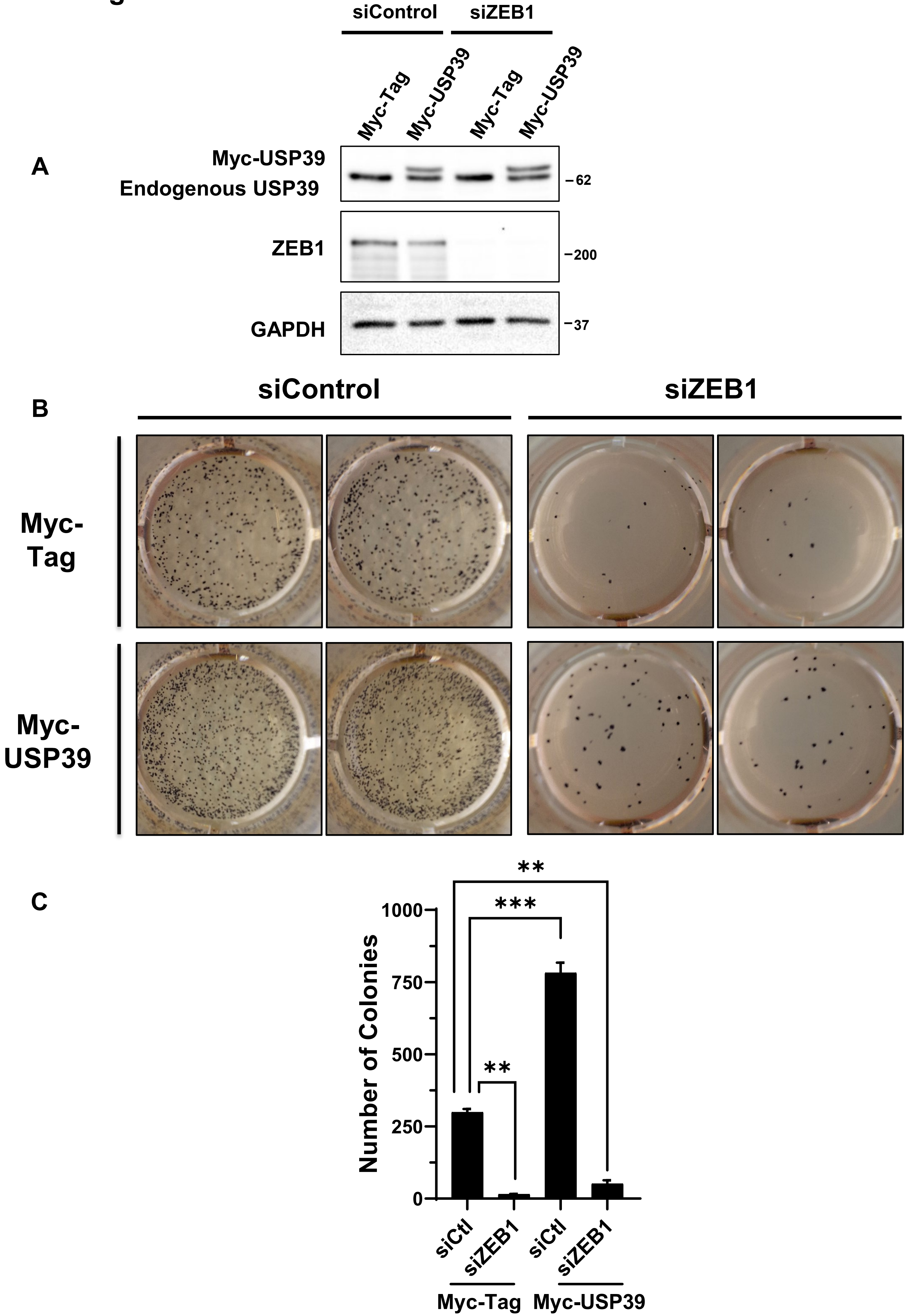
A
siControl
siZEB1
Myc-Tag
Myc-USP39
Myc-Tag
Myc-USP39
Myc-USP39
Endogenous USP39
ZEB1
GAPDH
62
200
37
B
siControl
siZEB1
Myc-Tag
Myc-USP39
C
Number of Colonies
1000
750
500
250
0
**
***
**
siCtl
siZEB1
siCtl
siZEB1
Myc-Tag
Myc-USP39

Figure S13

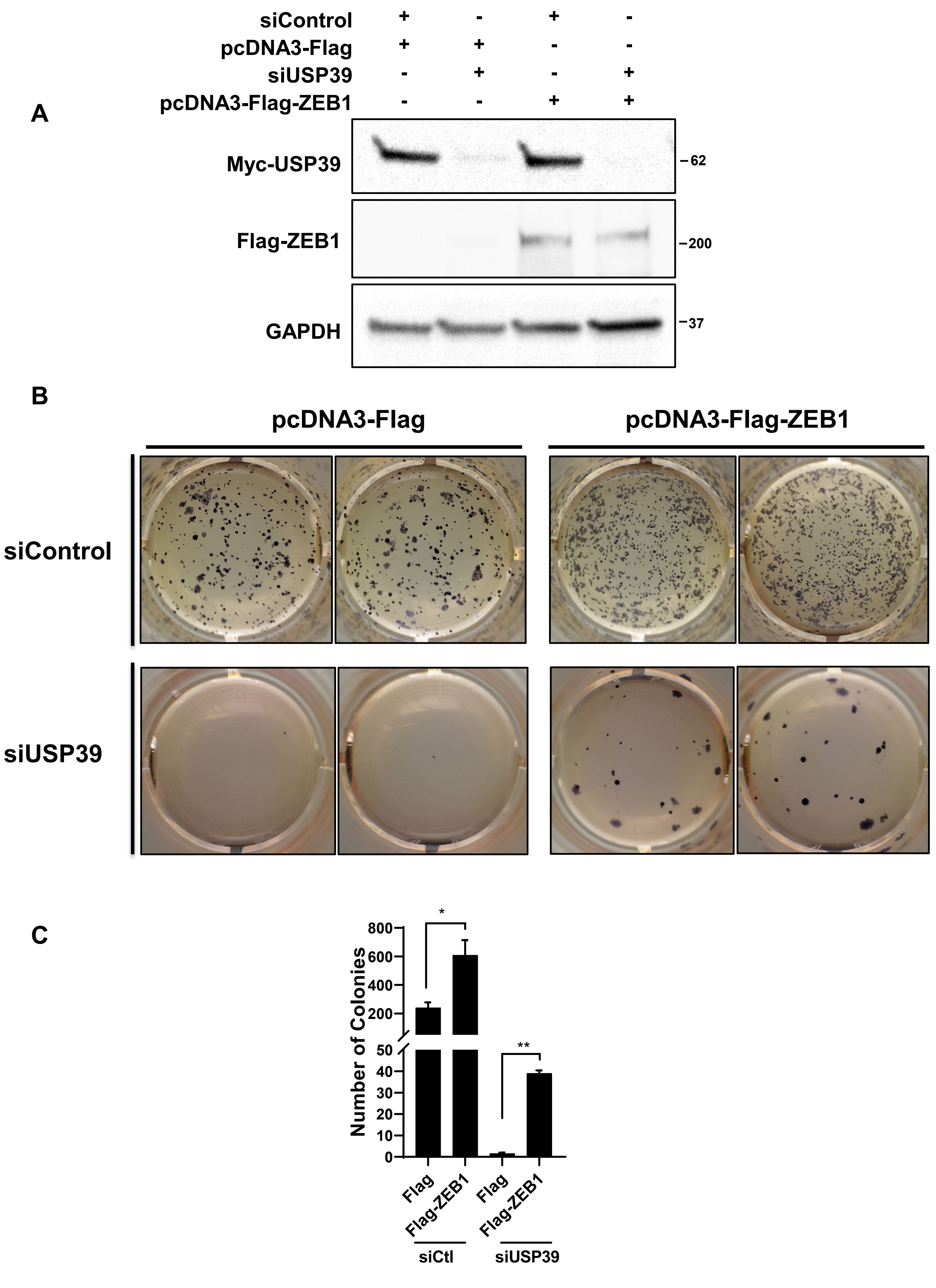
A
siControl + - + -
pcDNA3-Flag + + - -
siUSP39 - + - +
pcDNA3-Flag-ZEB1 - - + +
Myc-USP39
62
Flag-ZEB1
200
GAPDH
37
B
pcDNA3-Flag
pcDNA3-Flag-ZEB1
siControl
siUSP39
C
Number of Colonies
800
600
400
200
50
40
30
20
10
0
*
**
Flag
Flag-ZEB1
Flag
Flag-ZEB1
siCtl
siUSP39

Figure S14

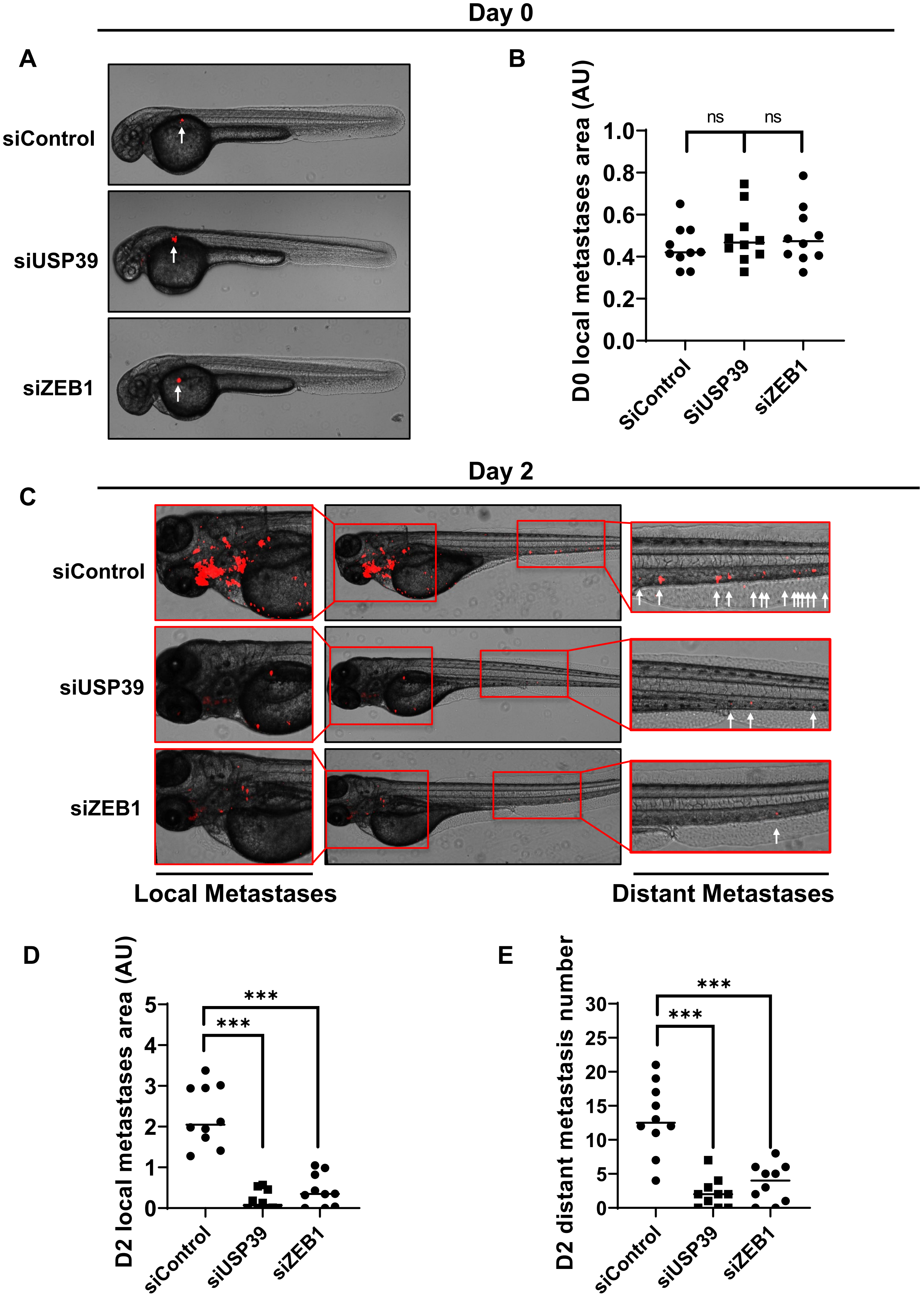

Day 0
A
siControl
siUSP39
siZEB1
B
D0 local metastases area (AU)
ns
ns
1.0
0.8
0.6
0.4
0.2
0.0
SiControl
SiUSP39
siZEB1
Day 2
C
siControl
siUSP39
siZEB1
Local Metastases
Distant Metastases
D
D2 local metastases area (AU)
***
***
5
4
3
2
1
0
siControl
siUSP39
siZEB1
E
D2 distant metastasis number
***
***
30
25
20
15
10
5
0
siControl
siUSP39
siZEB1

SUPPLEMENTAL MATERIAL AND METHODS

IHC

Immunohistochemistry for CD138 (#760-4248, Roche diagnostics, F. Hoffmann-La Roche, Basel, Switzerland) were performed using Myeloma tissue array (BM291d and BM483b) purchased from TissueArray.com. Antigen retrieval was performed by boiling sections for 10 min in citrate buffer (pH 6.0) and cooling at RT°, followed by blocking of endogenous peroxidase activity with 0.3% H_2O_2 for 30 min. The sections were blocked with 2.5% horse serum in TBS solution for 30 min in a humid chamber prior to incubation with anti-USP39 antibody (1/200). Positive cells were detected using an ImmPRESS HRP anti-rabbit detection kit. The immune complexes were visualized using a Peroxidase Substrate DAB kit (Vector) according to the manufacturer's protocol, and slides were counterstained with hematoxylin.

qPCR

Total RNA was isolated by using the RNeasy Plus Mini kit (QIAGEN, Hilden, Germany) and quantified by using a Nanodrop 2000 UV visible spectrophotometer. One microgram of mRNA was reverse-transcribed into cDNA by using an QuantiTect Reverse Transcription Kit (QIAGEN), according to the manufacturer's instructions. Real-time qPCR was performed on cDNA by using StepOnePlus™ Real-Time PCR System (Applied bioscience) and Takyon™ ROX SYBR 2X MasterMix dTTP blue (Eurogentec). Predesigned primer were purchased from Eurogentec (USP39 forward: TTG-GAA-GAG-GCG-AGA-TAA; USP39 reverse: AGG-AGC-ATC-AAT-CAT-CAT-C) and (ZEB1 forward: CAG-CTT-GAT-ACC-TGT-GAA-TGG-G; ZEB1 R: TAT-CTG-TGG-TCG-TGT-GGG-ACT). Fold changes in expression were calculated by the delta Ct method using RPLP0 primers (36B4 forward 5' GGC-CAG-GAC-TCG-TTT-GTA-CC; 36B4 R 5' CAGATTGGCTACCCAACTGTT) gene as an endogenous control for mRNA expression. All fold changes were expressed as normalized to the untreated control. Measurements were done in triplicate.

SUPPLEMENTAL FIGURE LEGENDS

Figure S1: CD138 expression in bone marrow of MM patients. (A, B) Representative pictures of CD138 staining from BM tissue sections of healthy donors **(A)** and MM patients **(B)**.

Figure S2: USP39 is widely expressed in MM cell lines. (A) Graph represents the mRNA expression levels of different MM cell lines from Depmap portal website. **B)** Cell lysates from different MM cell lines were subjected to immunoblot using USP39 and HSP90 antibodies.

Figure S3: **USP39 increases the clonogenic capacity of MM cells.** OPM2 and KMM1 cells were stably transfected with lentiviral particles encoding Myc or Myc-USP39 and were subjected to immunoblots using Myc and GAPDH antibodies **(A and B)**. The clonogenic capacity of the cells was measured after 10 days within a semi-solid medium. Representative pictures were shown in **B and D** and the quantification of the assay was reported in **C and E**.
Differences between the Myc and Myc-USP39 were analysed using the Unpaired Student's t-test. Statistical significance was denoted as follows: *P < 0.05, **P < 0.01, ***P < 0.001, ****P < 0.0001, NS (non-significant).

Figure S4: USP39 overexpression does not protect MM cells from BTZ treatment. U266 **(A)** and KMM1 **(B)** cells stably transfected with lentiviral particles encoding Myc or Myc-USP39 were stimulated with increased concentrations of BTZ (1, 3, 10, 30 and 100 ng/ml) for 24 hours and cell metabolism was measured by XTT assay.

The results are presented as the mean of at least three independent experiments ± SD. Statistical analysis was performed using ordinary one-way ANOVA to compare differences among all the experimental groups. Statistical significance is denoted as follows: *P < 0.05, **P < 0.01, ***P < 0.001, ****P < 0.0001, NS (non-significant).

Figure S5: USP39 depletion decreases the expression of ZEB1 over the time. The graphs illustrate the quantification of proteins detected by immunoblot, as shown in **Figure 5A**.
The results are presented as the mean of at least three independent experiments ± SD. Statistical analysis was performed using ordinary one-way ANOVA to compare differences among all the experimental groups. Statistical significance is denoted as follows: *P < 0.05, **P < 0.01, ***P < 0.001, ****P < 0.0001, NS (non-significant).

Figure S6: Quantification of USP39 protein following its overexpression or inhibition, in the presence or absence of CHX. The graphs illustrate the quantification of USP39 proteins detected by immunoblot, as shown in **Figure 6C and D**.
The results are presented as the mean of at least three independent experiments ± SD. Statistical analysis was performed using ordinary one-way ANOVA to compare differences among all the experimental groups. Statistical significance is denoted as follows: *P < 0.05, **P < 0.01, ***P < 0.001, ****P < 0.0001, NS (non-significant).

Figure S7: Depletion of USP39 does not decrease ZEB1 mRNA level. OPM2 cells were transfected with Control, USP39 or ZEB1 siRNA for 72h. Then, mRNA levels were measured by q-PCR.
The results are presented as the mean of at least three independent experiments ± SD. Statistical analysis was performed using ordinary one-way ANOVA to compare differences among all the experimental groups. Statistical significance is denoted as follows: **P < 0.01, ***P < 0.001.

Figure S8: USP39 Stabilizes and Deubiquitinates ZEB1 Protein The graphs illustrate the quantification of Ub-ZEB1 and ZEB1 proteins detected by immunoblot, as shown in **Figure 6G and 6H**.
Differences between the Empty and Myc-USP39 **(A)** or siControl and siUSP39 **(B)** were analysed using the Unpaired Student's t-test. Statistical significance was denoted as follows: *P < 0.05, **P < 0.01, ***P < 0.001, ****P < 0.0001, NS (non-significant).

Figure S9: Nuclear localization of USP39 in OPM2 cells. Fluorescence microscopy images of OPM2 cells stably infected with lentiviral particles encoding GFP (top) or GFP-USP39 (bottom) fusion proteins. The right panels represent a 10x magnification.

Figure S10: ZEB1 depletion diminishes in vitro cell proliferation, clonogenicity and induces late apoptosis in OPM2 and KMM1 MM cells. (A-E) OPM2 cells were transfected with either control, USP39 or ZEB1 siRNA for 72h. Then, lysates from these cells were subjected to immunoblots using GAPDH, USP39 and ZEB1 antibodies **(A)**. In parallel, the cell metabolism was assessed by XTT assay at 72h **(B)** and the percentage of cell death was measured by flow cytometry after IP staining **(C)**. Clonogenic capacity of the cells was measured after 10 days of transfection within a semi-solid medium **(D-E)**. Representative pictures were shown in **(D)** and the quantification of the assay was reported in **(E)**. KMM1 were treated as described for OPM2 cells and subjected to the same analysis **(F-I).**
The results are presented as the mean of at least three independent experiments ± SD. Statistical analysis was performed using ordinary one-way ANOVA to compare differences between the sicontrol and the other two groups or among all the experimental groups **(E)**. Statistical significance is denoted as follows: *P < 0.05, **P < 0.01, ***P < 0.001, ****P < 0.0001, NS (non-significant).

Figure S11: BTZ exacerbates the effect of ZEB1 depletion. OPM2 cells were transfected with either control, USP39 or ZEB1 siRNAs for 72 hours. Then cells were stimulated with increased concentrations of BTZ (3, 10, 30 ng/ml) for 24 hours and cell metabolism was measured by XTT assay.
The results are presented as the mean of at least three independent experiments ± SD. Statistical analysis was performed using ordinary one-way ANOVA to compare differences among all the experimental groups. Statistical significance is denoted as follows: *P < 0.05, **P < 0.01, ***P < 0.001, ****P < 0.0001, NS (non-significant).

Figure S12: Depletion of ZEB1 inhibits the enhancement in clonogenic capacity that is associated with USP39 overexpression. OPM2 cells stably transfected with lentiviral particles encoding Myc or Myc-USP39 were subjected to immunoblots using USP39, ZEB1 and GAPDH antibodies **(A)**. The clonogenic capacity of the cells was measured after 10 days within a semi-solid medium. Representative pictures were shown in **(B)** and the quantification of the assay was reported in **(C)**.
The results are presented as the mean of at least three independent experiments ± SD. Statistical analysis was performed using ordinary one-way ANOVA to compare differences between the sicontrol Myc-Tag and the other three groups or among all the experimental groups (E). Statistical significance is denoted as follows: *P < 0.05, **P < 0.01, ***P < 0.001, ****P < 0.0001, NS (non-significant).

Figure S13: Exogenous ZEB1 expression partially restored the clonogenic capacity of OPM2 cells that had been previously depleted for USP39. KMM1 cells were either transfected with Control or USP39 siRNAs for 24 hours. Then cells were transfected with Flag-Tag or Flag-ZEB1 vectors. After 72 hours, lysates from these cells were subjected to immunoblots using USP39, Flag-USP39 or GAPDH antibodies **(A)**. In parallel, clonogenic capacity of the cells was measured after 10 days of transfection within a semi-solid medium **(B-C)**. Representative pictures were shown in **(D)** and the quantification of the assay was reported in **(E)**.
The results are presented as the mean of at least three independent experiments ± SD. Statistical analysis was performed using ordinary one-way ANOVA to compare differences among all the experimental groups. Statistical significance is denoted as follows: *P < 0.05, **P < 0.01, ***P < 0.001, ****P < 0.0001, NS (non-significant).

Figure S14: The inhibition of USP39 and ZEB1 diminish the pro-metastatic capacity of myeloma cells. (A-E) (OPM2-Myc-USP39) were transfected during 48 hours with either control, USP39, or ZEB1 siRNA and subsequently injected into Zebrafish embryos (n=10) (labeled with red DiD) into the perivitelline space. Zebrafish embryos were monitored at Day 0 and Day 2 for tumor metastases using a fluorescent microscope. **(A)** Representative images of local metastases at day 0 are shown. **(B)** Quantification of the area of local metastases at Day 0. **(C)** Representative images of local and distal metastasis at day 2 are shown. Arrows indicate distant metastases. **(D)** Quantification of the area of local metastases at Day 2. **(E)** Quantification of the number of distant metastases at Day 2.
The Mann-Whitney U test was performed to compare the average measurement between siControl, siZEB1 and siUSP39 embryos. (U = 151, *P < 0.05, **P < 0.01, ***P < 0.001, ****P < 0.0001, NS (non-significant).
